# From Public Mental Health to Community Oral Health: The Impact of Dental Anxiety and Fear on Dental Status

**DOI:** 10.3389/fpubh.2014.00016

**Published:** 2014-02-28

**Authors:** Antonio Crego, María Carrillo-Díaz, Jason M. Armfield, Martín Romero

**Affiliations:** ^1^Department of Psychology, Madrid Open University (Udima), Madrid, Spain; ^2^Department of Paediatric Dentistry, Rey Juan Carlos University, Madrid, Spain; ^3^Australian Research Centre for Population Oral Health, University of Adelaide, Adelaide, SA, Australia

**Keywords:** dental anxiety, oral health, cognitive vulnerability model, access to health services, community dentistry

## Abstract

Dental fear is a widely experienced problem. Through a “vicious cycle dynamic,” fear of dental treatment, lower use of dental services, and oral health diseases reinforce each other. Research on the antecedents of dental anxiety could help to break this cycle, providing useful knowledge to design effective community programs aimed at preventing dental fear and its oral health-related consequences. In this regard, frameworks that analyze the interplay between cognitive and psychosocial determinants of fear, such as the Cognitive Vulnerability Model, are promising. The onset of dental fear often occurs in childhood, so focusing on the child population could greatly contribute to understanding dental fear mechanisms and prevent this problem extending into adulthood. Not only can public mental health contribute to population health, but also community dentistry programs can help to prevent dental fear. Regular dental visits seem to act in a prophylactic way, with dental professionals playing an important role in the regulation of the patients’ anxiety-related responses. Both public mental health and community dentistry could therefore benefit from a multidisciplinary approach to dental fear and oral health.

Mental disorders and subclinical psychological problems may influence physical health, acting as a barrier to seeking needed treatment. Conversely, physical conditions appear to increase the risk of suffering from mental illness (1–5). This “vicious cycle dynamic” has been also found in dental fear problems. Dental anxiety can block normal access to health services, which increases the risk of experiencing oral health diseases, and the progressive worsening of untreated oral symptoms can in turn reinforce the fear of dental procedures.

We argue that dental fear should be a matter of concern for public mental health, as this problem has a high prevalence in both adult and child populations and deleterious health-related consequences. Drawing from previous research, insights into the interplay between public mental health and community dentistry are provided. From our point of view, a multidisciplinary approach could be useful to prevent dental fear and promote oral health in child and adult populations, objectives which are connected to one another.

## Prevalence of Dental Fear and Anxiety in Child and Adult Populations

Figures for dental fear prevalence vary greatly. While some studies have reported percentages of adults with dental fear ranging between about 5 and 7% (6, 7), other surveys have reported considerably higher prevalence rates. For example, 24.3% of Dutch adults were found to have dental fear (8), 16.1% of the Australian population older than 5 years reported high levels of dental fear (9), and rates of extreme dental fear varied between 6 and 14% in a study carried out among Japanese university students (10).

The variation in prevalence rates across different studies could be a consequence of the diversity of measures of dental fear, methods, and study populations, but they could also reflect discrepancies in the criteria used to define dental fear and anxiety (8, 11). One study reporting results from a representative sample of the Australian adult population used the Index of Dental Fear and Anxiety, which allowed the calculation of several anxiety-related scores using differing diagnostic criteria. According to this research, 0.9% of surveyed people met DSM-IV criteria for a diagnosis of dental phobia, 2.2% suffered dental phobia if the criteria referred to acknowledging one’s fear as excessive or irrational were omitted, and 4.9% of respondents experienced a phobic disease with a dental component (12). Spanish research using the same instrument found similar results in a convenience sample, with 1.7% of participants meeting criteria for a DSM-IV diagnosis of specific dental phobia, 3.5% meeting less restrictive criteria of dental phobia, and 5.2% presenting phobia or disorder with a dental component (13).

The prevalence of dental fear in children is even more difficult to establish precisely. For instance, Rantavuori found prevalence rates varying from 6 to 56% depending on the study’s characteristics (14). Klingberg and Broberg, identified a narrower range, finding percentages of dentally fearful children ranging from 5.7 to 19.5%, depending on child age, with an overall mean of 11.1% (15). Previous research has also frequently reported figures within this range. For instance, high levels of dental fear have been found to be present in 7.6% of French children aged 5–11 years (16), 7.1% of British children aged 13–14 years (17), and 6.7% of Swedish children aged 4–11 years (18). A series of studies carried out in Spain has reported percentages between 4.9 and 13.6% in samples including participants aged from 7 through to 18 years (19–22).

Summarizing the existing literature, we currently lack a precise estimate of the percentage of people affected by dental fear and anxiety; however, a relatively high percentage of child and adult populations apparently suffer from high dental anxiety.

## The Vicious Cycle Dynamics Connecting Dental Anxiety and Oral Health Diseases

Dental fear has been argued to represent a major barrier to the delivery of oral health services. Several researchers have identified the existence of “vicious cycle dynamics” (23, 24) whereby dental fear is associated with a lower or more irregular frequency of dental visits (17, 21, 25–30), resulting in a worsening oral health status (30–33) and the subsequent reinforcement of dental treatment-related anxiety.

As dentally anxious patients are reluctant to seek dental care, they rarely benefit from preventive actions provided by regular check-ups. Furthermore, current oral pathologies of low or medium severity frequently remain untreated. In the absence of adequate dental treatment, oral symptoms will inevitably worsen, resulting in more severe oral health problems (30–33), which often require more intensive, urgent, and expensive treatments. Many dentally fearful patients will only attend the dentist when dental care is unavoidable, due to experienced pain or the intensity of symptoms. Not surprisingly, patients with more severe oral pathologies are more likely to receive potentially aversive dental treatments, which would confirm the patients’ anticipated negative expectations of dental visits and reinforce the avoidance pattern associated with dental anxiety (23, 24).

The “vicious cycle mechanism” can also be seen among children, with dental fear, attempts to escape from or avoid dental treatments, and poorer oral health status also associated in this population. Children with high dental anxiety have been found to visit the dentist less frequently (25, 26), and they also experience more untreated caries, worse periodontal condition, a higher number and probability of missing teeth, and greater need of oral rehabilitation (16, 27, 28, 34–36).

## Public Mental Health Meets Community Dentistry

Public mental health can contribute significantly to population oral health. As discussed, dental fear can act as a hindrance to dental care delivery, and this psychological problem can eventually turn into a health concern. In particular, addressing dental fear problems in child populations seems a priority task, due not only to its emotional- and health-related consequences, but also because child dental fear is frequently the precursor to dental fear problems in adulthood. Locker et al., for instance, found that approximately 50% of dental fearful individuals indicated that their fear developed in childhood, whereas 27 and 23% reported that their fear originated in adolescence and adulthood, respectively (37). More strikingly, Berggren and Meynert found that dental anxiety problems started in childhood for 85.3% of adults attending a specialized community dental unit for dental fear treatment (31).

Research has shown that dental treatment-related cognitive elements, i.e., thoughts, beliefs, assessments, and expectations about dental care delivery, constitute a major influence on dental fear (38–42). Indeed, cognitive elements have been found to be the best predictors of a person’s level of dental fear as compared with other variables connected with this problem (43). The Cognitive Vulnerability Model, in particular, has identified a cognitive schema, which comprises four inter-related assessments associated with dental fear (44). According to this framework, dentally fearful people assess dental events as potentially dangerous, uncontrollable, unpredictable, and disgusting. Overall, research conducted using this model has supported the idea of a cognitive vulnerability schema operating in dental fear in both adults (45, 46) and children (29, 47), with cognitive vulnerability perceptions accounting for just under one-half of the variance in dental fear scores beyond that accounted for by demographic variables and aversive dental experiences (45). However, while negative thoughts about dental treatments are clearly a strong risk factor for both dental fear and poorer oral health, we still do not know the extent to which these cognitions are present in the population, and this remains a task for research in public mental health.

Family-related variables also seem to play a role in both dental anxiety and fearful dental cognitions. Parental and child dental fear have been found to be consistently associated (48). Children’s fearful cognitions are connected with those of their parents, and parents’ cognitive vulnerability levels are significant predictors of children’s dental fear (46). Public mental health programs are suggested to address the interplay between cognitive assessments and family-related issues, in order to reduce children’s levels of dental fear.

While community dentistry could benefit from public mental health involvement, population mental health, in relation to dental fear in particular, could be improved by dental professionals’ work. For instance, regular check-ups could help provide children with positive or neutral dental treatment-related experiences. It is generally regarded that frequent attendance to the dentist fosters familiarity with dental procedures and helps to prevent future dental anxiety problems (41, 49, 50), playing a prophylactic role concerning the development of dental fear and children.

## Challenges for Dental Fear Research in a Public Health Context

Previous research has shown that dental anxiety is not only a mental health issue but also a public health concern. Fear of dental treatment increases the likelihood of experiencing oral health problems as a result of irregular or delayed dental visiting, and severe oral symptoms may reinforce dental fear, as potentially disgusting or threatening interventions are anticipated.

As discussed above, dental phobia and subclinical dental fear-related problems are relatively common in the population. However, more precise data on the prevalence of dental fear-related problems are required, which could help to identify groups at risk of experiencing this problem. Epidemiological studies should pay attention to both dental phobia and subclinical forms of this problem, otherwise relevant information will be missed (51). People with moderate/high levels of dental fear and anxiety, even if not meeting DSM-5 criteria for a dental phobia diagnosis, can enter into the “vicious cycle” of dental fear and be reluctant to regularly visit the dentist.

Breaking the vicious cycle of fear and anxiety requires a multidisciplinary approach, which involves both mental health and community dentistry professionals. From our point of view, research in this field faces two main challenges. First, better knowledge of the antecedents of dental anxiety are needed in order to design effective community programs aimed at preventing dental fear and its oral health-related consequences. In particular, the interplay between cognitive and psychosocial determinants of dental fear appears as a promising area of enquiry, as these factors could be targeted at a population level. The Cognitive Vulnerability Model, which focuses on dangerousness, uncontrollability, unpredictability, and disgustingness appraisals, offers a compelling framework to analyze these relationships (47). Second, studies focused on children could help to deepen our understanding of the mechanisms involved in dental fear development, as this problem usually emerges in childhood and subsequently extends to adulthood. In this regard, focusing on children’s dental anxiety could be a smart strategy to prevent adults’ fear.

## Conflict of Interest Statement

The authors declare that the research was conducted in the absence of any commercial or financial relationships that could be construed as a potential conflict of interest.
